# The American Electro-Therapeutic Association

**Published:** 1891-02

**Authors:** Wm. H. Walling

**Affiliations:** Secretary, 2005 Arch St., Philadelphia, Pa.


The American Electro-Therapeutic Association was organized on the 2d of January, 1891, at the Academy of Medicine, No 17 West 43d St., New York, by the adoption of a Constitution and By-Laws, and the election of the following officers: President, G. Betton Massey, M. D., Philadelphia; Vice-Presidents, William James Morton, M. D., and Augustin H. Goelet, M. D., New York. Secretary, William H. Walling, M. D., Philadelphia. Treasurer, George H. Rohe, M. D., Baltimore.

Executive Council: Horatio R. Bigelow, M. D., of Philadelphia, Franklin H. Martin, M. D., Chicago, William F. Hutchinson, M. D., Providence, R. I., Frederick Peterson, M. D., New York, and Chauncey DePalmer, M. D., Cincinnati, O.

The object of the Association, as stated in article second of the Constitution, is "The cultivation and promotion of knowledge in whatever relates to the application of electricity in medicine and surgery."

The Association starts with a strong and vigorous membership, and has every prospect of a most useful and successful career.

The next meeting will be held in Philadelphia, in September, of this year. Wm. H. Walling, M. D., Secretary,
2005 Arch St., Philadelphia, Pa.

